# Identification of immune-related proteins of *Dreissena polymorpha* hemocytes and plasma involved in host-microbe interactions by differential proteomics

**DOI:** 10.1038/s41598-020-63321-z

**Published:** 2020-04-10

**Authors:** Maxime Leprêtre, Christine Almunia, Jean Armengaud, Antoine Le Guernic, Arnaud Salvador, Alain Geffard, Mélissa Palos-Ladeiro

**Affiliations:** 1 Université de Reims Champagne-Ardenne, UMR-I 02 INERIS-URCA-ULH SEBIO Stress Environnementaux et BIOsurveillance des milieux aquatiques, UFR Sciences Exactes et Naturelles, Campus du Moulin de la Housse, BP 1039, 51687 Reims, CEDEX France; 2 Laboratoire Innovations Technologiques pour la Détection et le Diagnostic (Li2D), Service de Pharmacologie et Immunoanalyse (SPI), CEA, INRA, F-30207 Bagnols-sur-Cèze, France; 3 Université de Lyon, Université Claude Bernard Lyon 1, Institut des Sciences Analytiques, CNRS UMR 5280, F-69100 Villeurbanne, France

**Keywords:** Antimicrobial responses, Innate immunity, Proteomics

## Abstract

Biological responses of zebra mussel *Dreissena polymorpha* are investigated to assess the impact of contaminants on aquatic organisms and ecosystems. In addition to concentrate chemical contaminants in their tissues, zebra mussels accumulate several microorganisms such as viruses, protozoa and bacteria. In order to understand the molecular mechanisms involved in the defence against microorganisms this study aims at identifying immune proteins from *D. polymorpha* hemolymph involved in defence against protozoa and viruses. For this purpose, hemolymph were exposed *ex vivo* to *Cryptosporidium parvum* and RNA poly I:C. Differential proteomics on both hemocytes and plasma revealed immune proteins modulated under exposures. Different patterns of response were observed after *C. parvum* and RNA poly I:C exposures. The number of modulated proteins per hemolymphatic compartments suggest that *C. parvum* is managed in cells while RNA poly I:C is managed in plasma after 4 h exposure. BLAST annotation and GO terms enrichment analysis revealed further characteristics of immune mechanisms. Results showed that many proteins involved in the recognition and destruction of microorganisms were modulated in both exposure conditions, while proteins related to phagocytosis and apoptosis were exclusively modulated by *C. parvum*. This differential proteomic analysis highlights in zebra mussels modulated proteins involved in the response to microorganisms, which reflect a broad range of immune mechanisms such as recognition, internalization and destruction of microorganisms. This study paves the way for the identification of new markers of immune processes that can be used to assess the impact of both chemical and biological contaminations on the health status of aquatic organisms.

## Introduction

The Zebra mussel, *Dreissena polymorpha*, is a relevant sentinel species used in aquatic ecotoxicology, due to its ability to accumulate and concentrate the chemical and biological contaminants^1^. While most ecotoxicological studies focus on the impact of chemical contamination on aquatic organisms, few studies assess the impact of the biological contamination. However, recent studies have shown the ability of zebra mussels to bioaccumulate and concentrate several microorganisms. Gu and Mictchell^2^ considered the bivalve *D. polymorpha* as a reservoir of opportunistic pathogenic microorganisms to aquatic organisms and humans. Bioaccumulation of several protozoa pathogenic to humans in the zebra mussel tissues have been evidenced by numerous studies^3^. Furthermore, Mezzanotte *et al*.^4^ have demonstrated the ability of *D. polymorpha* to remove enteric viruses and the bacteria *Escherichia coli* found in municipal treated effluent. While literature proved the accumulation of several microorganisms in the tissues of zebra mussel, their interactions with the physiology of *D. polymorpha* remain to be clarified.

Probably more than other biological processes, the innate immune defence of bivalves is directly involved in the interaction with microbes. Indeed, the immune system acts as the first line of defence against microorganisms, involving physical barriers, phagocytic cells and a variety of immune effectors^5^. Innate immune system process can be summarized in three main steps: (i) the recognition of molecular motifs associated with microorganisms (MAMPs, Microbe-associated molecular patterns) or endogenous molecules secreted by damaged tissues (DAMPs, Damage-associated molecular patterns) by soluble compounds and cellular receptors, (ii) the activation of different signalling pathways, (iii) the production of molecular effectors involved in host defence and cellular defence responses^5^. In bivalves, the immune defence is mainly undertaken by their circulating fluid named hemolymph, which includes circulating immune cells, the hemocytes, and an acellular fraction, the plasma. A cross-talk between hemocytes and plasma appears crucial for an effective immune response^5^. In addition to their major role in phagocytosis of microbes, hemocytes produce and release a wide range of humoral factors that control cell-mediated responses and fight off microbial invaders. In plasma, secreted proteins work as the first line defence against microbes since they actively participate in recognition and destruction of microorganisms as well as in immune signaling^6^. Interaction between the immune system of bivalves and microorganisms have been mainly investigated in marine bivalves due to their economic interests and the high number of infectious diseases affecting aquaculture farms^7^. These studies resulted in the identification of several cellular and molecular immune mechanisms involved in the defence against important pathogens. While anti-bacterial responses are well documented in bivalves, little is known on anti-protozoal responses and the anti-viral defence^5^. In addition, immune responses of bivalves are generally investigated in the framework of challenges with their related pathogens. Indeed, few studies investigated the biological interactions with non-pathogenic microbes which could be found in water courses. Furthermore, in contrast with marine bivalves, interactions between the immune system of freshwater bivalves and microorganisms are rarely investigated despite their interest in ecotoxicological studies. For *D. polymorpha*, information regarding modulation of the immune system by biological contamination is relatively scarce. However, the limited literatures reported that microorganisms that interact with *D. polymorpha* modulate cellular mediated immune responses of hemocytes. Juhel *et al*.^8^ observed a decrease in total hemocytes count, a modulation of phagocytic rate of hemocytes and an increase of the concentration of lysozymes in mussels fed with a microcystin-producing cyanobacterium. Recently, the interaction between three human protozoan parasites and *D. polymorpha* immunity was investigated at the cellular level^9,10^. Authors revealed the ability of protozoa to induce cytotoxic effects and modulate phagocytosis of *D. polymorpha* hemocytes. Moreover, involvement of apoptosis during host-parasite interactions have been evidenced in hemocytes of zebra mussels^11^. While the interactions between the immune system of *D. polymorpha* and microorganisms are investigated at the cellular level, the immune molecular mechanisms involved in host-microbe interactions are still not documented. Characterization of these interactions at the molecular level would expand our knowledge on microbe-bivalve interactions and more specifically on the innate immune system of *D. polymorpha*.

Next generation shotgun proteomics appears to be a useful methodology to discover and prioritize new biomarkers of contamination, while targeted proteomics allows the validation of these biomarkers for diagnostic purpose^12–14^. Recently, a proteogenomic analysis was performed on the hemocytes and plasma of *D. polymorpha*, resulting in the identification of more than 3,000 proteins^15^. In order to get further insights into the immune proteome of *D. polymorpha*, many proteins potentially involved in the recognition, internalization and destruction of microorganisms were pinpointed in both hemocytes and plasma fractions. Considering the high number of immune proteins observed in plasma, the need to consider both intracellular and extracellular fractions to investigate the immune defence occurring in the hemolymph compartments was pointed out. Nevertheless, this proteogenomic analysis was performed on zebra mussels that were physiologically acclimated to laboratory conditions and thus only gave a cartography of potential immune-related proteins without exploring their interactions with microbes.

The present study aims to identify proteins involved in the interaction between microorganisms and the immune system of *D. polymorpha*. For this purpose, *ex vivo* challenges were performed to decipher the immune system response of *D. polymorpha* upon protozoan and virus challenges. *D. polymorpha* hemolymph was exposed either to a protozoan, *Cryptosporidium parvum*, or a synthetic double stranded RNA (dsRNA) known to induce a non-specific antiviral immune responses in bivalves^16,17^. A comparative proteomic analysis was then performed on both hemocytes and plasma fractions to reveal immune-related proteins modulated by *C. parvum* and RNA polyinosinic-polycytidylic acid (poly I:C) exposures.

## Results and Discussion

### Nano LC-MS/MS strategy for the discovery of modulated proteins in hemocytes and plasma of *D. polymorpha*

**Figure 1:**
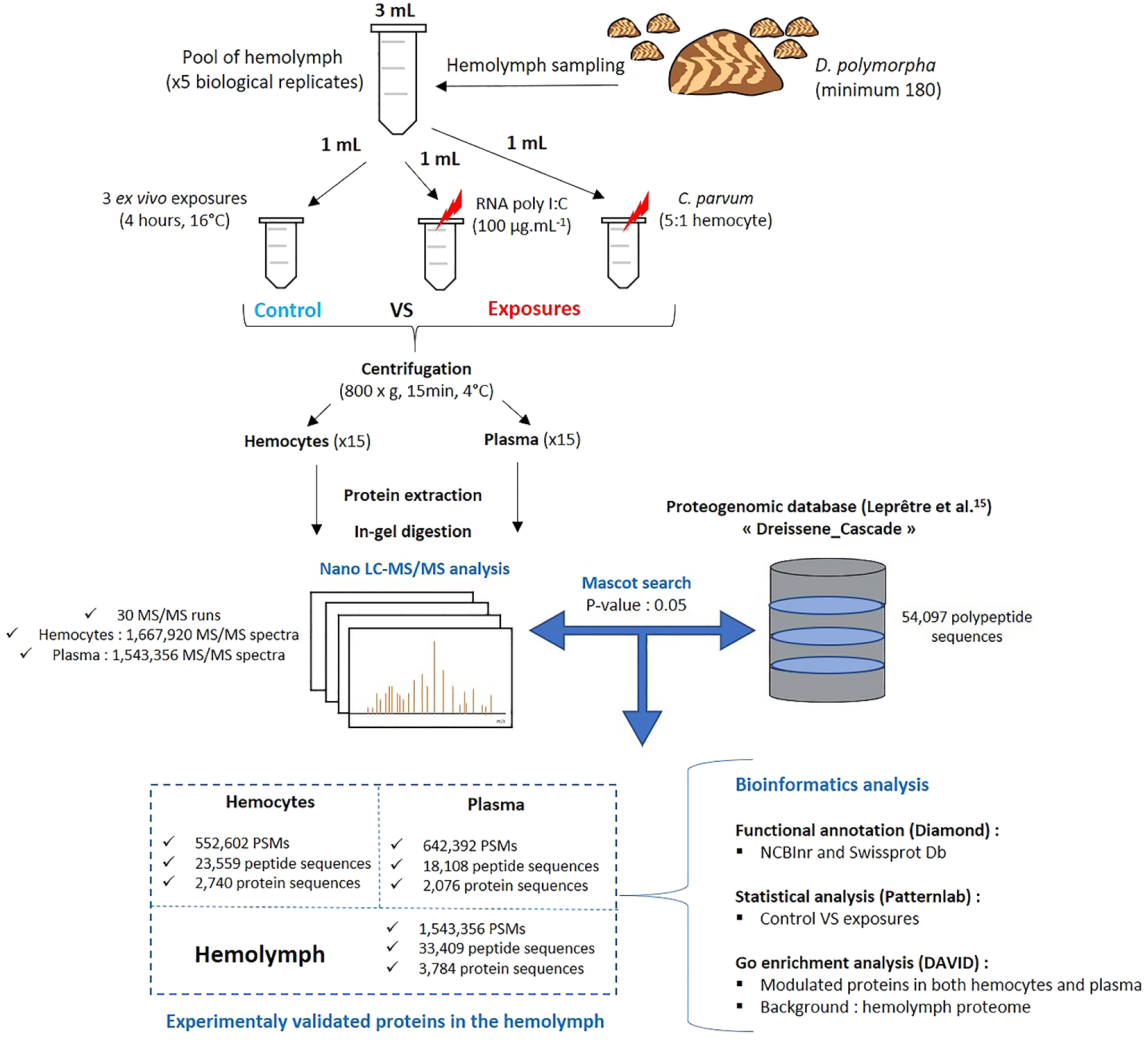
Experimental design. Schematic workflow of the protocol followed for the identification and quantification of proteins in the hemolymph of *Dreissena polymorpha*.

A differential proteomic analysis was conducted on hemocytes and plasma from the hemolymph challenged with immune modulators in order to identified proteins involved in the immune defence of *D. polymorpha*. Our strategy was to start from a unique pool of hemolymph to perform the three exposure conditions (Fig. 1). In this way, biological variability is minimized as much as possible and changes in protein abundance are more likely the results of exposures to *C. parvum* and RNA poly I:C. In total, 30 runs of LC-MS/MS analysis were performed, resulting in the recording of 1,667,920 MS/MS spectra from the 15 hemocyte samples and 1,543,356 MS/MS spectra from the 15 plasma samples. MS/MS spectra were assigned using the protein database “Dreissene_cascade” which contains 54,097 putative polypeptide sequences that have been prioritized with a first round of search with experimental MS/MS datasets as described in Leprêtre *et al*.^15^. From the whole dataset comprising 3,211,276 MS/MS spectra, a total of 1,198,994 MS/MS spectra (36%) were interpreted into peptide sequences, leading to the discovery of 33,409 unique peptides and detection of 3,784 proteins from all samples (hemocytes and plasma) (Fig. 1). Results of comparative proteomics reflect the proteome changes of *D. polymorpha* after four hours of exposure. A higher number of proteins were identified with at least 2 peptides in hemocytes (2,740) compared to plasma (2,076). From them, 1,032 proteins were common between plasma and hemocytes, 1,708 proteins were exclusive to hemocytes and 1,044 to plasma (Fig. 1). Regarding BLAST annotation, the sequence similarity search against NCBInr and Swissprot Databases resulted in the functional annotation of 3,656 proteins, letting about 3% of proteins without BLAST annotation. Once again, proteogenomic analysis has shown its efficiency in the quick identification of proteins from non-model species without genome sequence and annotation since more than 3,500 proteins could be identified in the hemolymph of *D. polymorpha*. As described in Leprêtre *et al*.^15^, a greater number of proteins were observed in hemocytes than in plasma, as expected as the dynamic ranges of protein abundance in cells and plasma are quite different in all animals. Furthermore, several proteins were found in both compartments, suggesting that they are secreted by hemocytes in the extracellular space. The enrichment of some proteins in the latter compartment indicates their longer half-life in this compartment or their origin that could differ.

**Figure 2:**
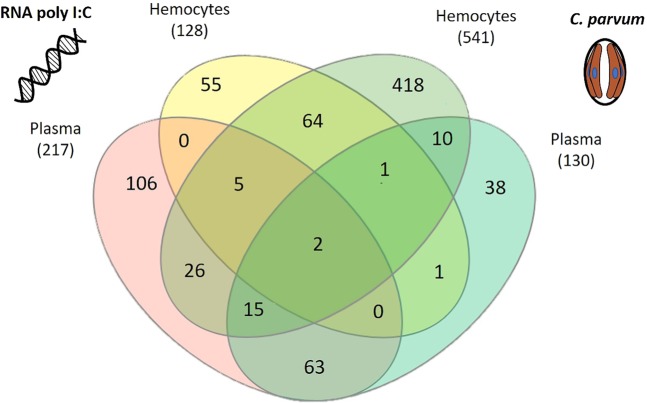
Venn diagram representing significantly modulated proteins in hemocytes and plasma challenged with RNA poly I:C and *C. parvum*. Numbers in brackets refer to the total number of proteins whose abundance is modulated significantly (p-value <0.05) in hemocytes or plasma challenge with *C. parvum* and RNA poly I:C.

The patternLab’s T-Fold module was used to pinpoint significantly modulated proteins in hemocytes or plasma from the hemolymph samples exposed to *C. parvum* or RNA poly I:C in comparison to the control condition. As five replicates per condition have been performed and analyzed with a robust methodology, the differential proteomics results are relevant. A total of 643 proteins were significantly modulated in the hemolymph exposed to *C. parvum* and 328 proteins were also significantly modulated in the hemolymph exposed to RNA poly I:C (Fig. 2). The abundance of several proteins was found modulated in both hemocytes and plasma exposed to biological stressors. Different patterns of modulation were observed between the hemolymph challenged with *C. parvum* and RNA poly I: C (Fig. 2). While in *C. parvum* exposure, a greater number of proteins were modulated in terms of abundance in hemocytes compared to plasma, the opposite was observed in the hemolymph exposed to RNA poly I:C, with a higher number of modulated proteins observed in plasma compared to hemocytes. Such results may suggest, that after four hours of exposure, *C. parvum* is managed in hemocytes while RNA poly I:C seems to be managed in the extracellular space. Several studies identified significant changes in the proteome of mollusks challenged with a protozoan. As our results, a higher number of proteins were modulated in hemocytes compared to plasma in the octopus, *Octopus vulgaris*, infected with the protozoan parasite *Aggregata octopiana* and the Manila clam, *Ruditapes philippinarum*, challenged with *Perkinsus olseni* using a 2D-gel electrophoresis methodology^18,19^. The internalization of *C. parvum* by hemocytes have been reported in the freshwater benthic clam *Corbicula fluminea* during *in vitro* and *in vivo* exposures^20^. Using flow cytometry, labeled *C. parvum* were endocytosed by zebra mussel hemocytes exposed 4 h in *ex vivo* condition^9^. In this case, the management of protozoa by immune cells involves a dynamic modulation of intracellular proteins to internalize and eliminate protozoa. For viral infection, molecular responses were till now mainly investigated in immune cells using transcriptomic analysis. However, the immune responses against viruses occurring in extracellular fraction is gaining attention. For example, a comparative proteomic analysis was performed in the plasma of the zebra fish to understand spring viremia of carp virus pathogenesis^21^. Infections of zebra fish resulted in the modulation of more than 150 proteins and provided the identification of multiple viral-infection biomarkers. In invertebrates, only Tao *et al*.^22^ performed a proteomic analysis on the plasma of shrimp *Litopeaneus vannamei* infected with the white spot syndrome virus (WSSV). Among the 486 proteins identified in the plasma, 59 proteins were modulated upon WSSV infection. Both studies pointed out the importance of protein responses occurring in the extracellular fluid during viral infections. Furthermore multifunctional plasma proteins from marine mollusks have been identified to have broad-spectrum antiviral activity against mammalian viruses^17^.

The Fig. 2 shows overlapped and specific proteins modulated in hemocytes and plasma exposed to *C. parvum* and RNA poly I:C. Only two proteins, a peroxiredoxin and a cysteine protease, were modulated in all conditions. In hemocytes, 418 proteins were exclusively modulated in *C. parvum* exposure and 55 proteins were exclusively modulated in the RNA poly I:C condition. In addition, 72 proteins were modulated in both treatments, which represent about 50% of the proteins modulated in hemocytes challenge with RNA poly I:C. In plasma, 106 proteins were modulated exclusively in the RNA poly I:C treatment against 38 proteins exclusively modulated in the *C. parvum* challenge. More than 50% of plasmatic proteins modulated by *C. parvum* were also modulated in the plasma of RNA poly I:C exposure. Overall, these results support that common and distinct biological processes are taking place between the hemolymph exposed to *C. parvum* and RNA poly I:C. While bivalves lack the mechanisms conferring adaptive immunity, recent research demonstrates that they possess a certain level of immune specificity depending on the structural composition and biological properties of microorganisms^5^. In our study, *C. parvum* was used in its oocyst form and RNA poly I:C is a synthetic dsRNA which mimic virus. Their difference in term of molecular structure probably requires an appropriate immune response for their elimination in *D. polymorpha* hemolymph, which could explain the different pattern of modulation observed.

### Identification of immune-related proteins involved in host-microbial interactions

A Gene set enrichment analysis (GSEA) was performed on significantly modulated proteins in the hemolymph exposed to *C. parvum* and RNA poly I:C with the aim to reveal biological processes modulated under these two exposure conditions (Table 1). Results showed that under both exposure conditions, several immune-related biological processes were identified, in particular those related to the cytoskeletal reorganization during endocytosis. In addition, BLAST annotation of significantly modulated proteins provided the identification of proteins involved in the defence against *C. parvum* and RNA poly I:C. Although these proteins can take part in multiple biological processes, they were categorized into one of the following immune functional groups: 1) pattern recognition receptors (PRRs), 2) immune signaling, 3) internalization of microorganisms, 4) immune effectors, 5) apoptosis or 6) other processes. While many of them were modulated in both exposure conditions (Table 2), some were modulated exclusively under *C. parvum* (Table 3) or RNA poly I:C challenges (Table 4).

**Table 1:** Immune-related biological processes enriched in the hemolymph challenged with *C. parvum* or RNA poly I:C. Count: number of modulated proteins related to BPs; Modulated protein: percentage of modulated proteins related to BPs; p-value: modified Fisher’ exact test p-value.

	Biological process (BPs)	Count	Modulated protein (%)	p-value
*C. parvum*	vesicle-mediated transport	84	16.4	1.8E-02
	cytoskeleton organization	70	13.6	1.8E-05
	locomotion	67	13.1	4.1E-02
	small GTPase mediated signal transduction	26	5.1	2.6E-02
	actin polymerization or depolymerization	23	4.5	8.7E-04
	Ras protein signal transduction	17	3.3	3.4E-02
	regulation of apoptotic signaling pathway	17	3.3	1.8E-02
	vesicle organization	16	3.1	2.5E-02
	establishment or maintenance of cell polarity	16	3.1	1.5E-02
	Arp2/3 complex-mediated actin nucleation	8	1.6	5.1E-02
	apoptotic cell clearance	8	1.6	5.1E-02
	lamellipodium assembly	7	1.4	3.6E-02
RNA poly I:C	response to stress	68	28.0	2.2E-03
	response to chemical	62	25.5	1.2E-02
	locomotion	42	17.3	6.2E-04
	biological adhesion	41	16.9	1.1E-03
	immune system process	40	16.5	3.3E-02
	cell adhesion	40	16.5	1.7E-03
	cytoskeleton organization	38	15.6	1.3E-04
	neurogenesis	31	12.8	1.6E-02
	negative regulation of hydrolase activity	12	4.9	3.1E-02
	humoral immune response	10	4.1	2.3E-02
	oxidoreduction coenzyme metabolic process	10	4.1	9.9E-03
	complement activation	8	3.3	5.0E-02

#### Immune proteins modulated in both exposures

The recognition of non-self molecules is an essential step to trigger an appropriate and an effective immune response. Immune responses are initiated by the recognition of MAMs or DAMPs by PRRS. Signal transduction pathways induced by PRRs result in the activation of gene expression and synthesis of a broad range of molecules involved in immune defence. Exposure of the hemolymph to *C. parvum* and RNA poly I:C resulted in the modulation of several potential PRRs (Table 2). “Among them, several lectins were” up-regulated in both exposures. Lectins are made of carbohydrate recognition domains which allow the recognition of non-self-carbohydrate domains located at the surface of microorganisms such as glycoproteins of the viral envelope or glycans of parasites^23^. The cation-independent mannose-6-phosphate receptor (M6PR) is a P-type lectin that was positively regulated in the plasma exposed to contaminants with a fold change (FC) higher than 1.6. In vertebrates, M6PRs are believe to play a critical role in the biogenesis of lysosomes by targeting hydrolytic enzymes to lysosomes^24^. Induction of M6PRs also plays an important role during viral infection, by facilitating the entry of viruses into cells^25,26^. In invertebrates, few studies have investigated the role of M6PRs in the immunity. Recently, Zhang *et al*.^27^ reported an induction of M6PRs genes in shrimp challenged with a broad range of bacteria species. Authors demonstrated that M6PR functioned as a pattern recognition receptor by binding to peptidoglycan (PGN), lipopolysaccharide (LPS), and lipoteichoic acid (LTA) in addition to their implication in antimicrobial peptides production. While involvement of M6PR in response to protozoa and viruses is still not investigated in bivalves, the up regulation of this protein observed in plasma exposed to *C. parvum* and RNA poly I:C might suggest its involvement in *D. polymorpha* immunity. Interestingly, a natterin-like protein was down-regulated (FC: −1.7) in hemocytes exposed to *C. parvum* and up-regulated in the plasma exposed to RNA poly I:C (FC: 1.3). Natterin-like protein is one of the most abundant protein of *D. polymorpha* hemolymph and is composed of a jacalin-lectin domain, which may confer an affinity with carbohydrate motifs found in microorganisms^15^. Natterins were first identified as the major toxin of the venomous fish *Thalassophryne nattereri* and were described as an important defence proteins against predators^28–30^. The implication of natterins in the immune defence of bivalves is still not investigated and its involvement in the recognition of microorganisms is not clearly demonstrated. However, a recent study showed that proteins structurally close to natterins serve as a multipotent pattern recognition receptor in the oyster immune defence^31^. The presence of jacalin-lectin and toxin domains in the natterin-like protein of *D. polymorpha* as well as its modulation pattern during exposures to *C. parvum* and dsRNA may indicate its implication in the *D. polymorpha* immune defence against microbes. Galactose binding lectins (galectins) and a Beta-1,3-glucan binding protein (BGP) were also modulated in the hemolymph exposed to protozoan and dsRNA. As reviewed in Vasta *et al*.^32^, galectins are non-self-recognition receptors involved in both innate and adaptive immune systems. In aquatic mollusks, the role of galectins in the defence against microorganisms is well documented^33^. It appears that galectins work as PRRs and interact with bacteria, protozoa and viruses. In innate immunity, BGP functions as a PRR that recognizes glucans of bacteria cell walls. To our knowledge, only Roux *et al*.^34^ have reported an overexpression of Lipopolysaccharide and B-1,3-Glucan Binding Protein (LBGP) gene in shrimp infected by virus. The authors hypothesized that in addition to their PRR functions, LBGP may play a critical role in viral pathogenesis. Oocyst wall of *Cryptosporidium* is composed of carbohydrates, proteins and lipids but lacks β-1,3-glucan polysaccharides^35,36^. However numerous oocyst walls of human pathogens contain β-1,3-glucan which may have affinity with BGP proteins^37^. In this way, BPG from *D. polymorpha* may play a role in the general anti-protozoal response. Proteins containing C1q domains were also highly modulated in plasma exposed to *C. parvum* and RNA poly I:C (Table 2). C1q proteins are highly diversified proteins in bivalves and work as PRRs that recognize a broad range of microorganisms and may trigger the activation of the complement pathways^38,39^.

**Table 2:** Immune related proteins significantly modulated in the hemolymph of *D. polymorpha* under both *C. parvum* and RNA poly I:C challenge. The BLAST annotation and expect value are derived from the sequence similarity search against NCBInr database. H = hemocytes and P = plasma and the fold change of significantly modulated proteins (p-value < 0.05) between treated and control sample is indicated.

BLAST annotation (NCBInr)			*C. parvum*		RNA poly I:C	
Immune proteins	E-value	Conserved domain	H	P	H	P
**Pattern recognition receptors (PRRs)**						
natterin-like protein	2.5E-09	jacalin-like lectin domain, C-terminal toxin domain	‒1.7	—	—	1.3
Cation-independent mannose-6-phosphate receptor (M6PR)	0.0E + 00	M6PR repeat domains	—	1.6	—	2
Galactose binding lectin	5.7E-19	Galactose binding lectin domain	1.5	‒1.6	—	‒1.9
Beta-1,3-glucan-binding protein	5.2E-99	cytolytic factor, Glycosyl hydrolase domain, beta-1,3-glucan recognition protein	—	−1.3	—	−1.3
**Immune signaling**						
Macrophage migration inhibitory factor (MIF)	1.2E-40	MIF domain	2.5	—	2.3	—
**Microorganism Internalization**						
myosin-2 essential light chain-like	2.9E-58	EF-hand, calcium binding motif, Ca2 + -binding protein	1.7	—	1.4	—
integrin beta-like protein	2.8E-140	Integrin beta chain VWA domain, Integrin plexin domain	−1.4	—	—	1.8
integrin alpha-9-like isoform X2	1.9E-54	integrin alpha domain	—	1.4	—	1.7
**Immune effectors**						
Antimicrobial peptides						
Histone H2A.V	1.3E-54	Core histone domain, C-terminus of histone H2A	6	—	4.6	—
Proteases, protease inhibitors and hydrolases						
cysteine proteinase	9.3E-145	Cathepsin propeptide inhibitor domain, Peptidase C1A subfamily	1.4	4.4	2.5	5.4
serine protease-like protein	7.4E-19	Trypsin-like serine protease domain	-	2.9	-	3.2
Metalloproteinase- like protein	2.9E-212	Zinc-dependent metalloprotease domain	−1.9	—	−1.6	—
cystatin B	1.5E-18	Cystatin domain	1.5	1.4	1.4	—
Sucrase-isomaltase	0.0E + 00	Glycoside hydrolases domains	—	1.4	—	2.2
Complement system						
complement C1q-like protein	2.3E-03	C1q domain	—	−1.7	—	−2.3
complement C1q-like protein	2.2E-13	C1q domain	—	1.4	—	1.5
Complement C1q-like protein	2.3E-03	C1q domain	—	−1.6	—	−1.7
complement factor B-like	2.8E-22	von Willebrand factor domain; Complement control protein (CCP)	—	−1.8	—	−1.6
complement C2	8.9E-16	Trypsin-like serine protease, Von Willebrand factor type A (vWA) domain	—	1.7	—	2.2
complement component C3	4.0E-24	Alpha-2-macroglobulin domain		1.7		1.5
complement component C3	1.5E-08	Alpha-2-macroglobulin domain		−2.9		−3.6
**Other immune-related process**						
26S protease regulatory subunit	7.3E-217	26S proteasome regulatory subunit	1.5		1.7	
Cytochrome c oxidase	1.2E-15	Cytochrome c oxidase subunit Va	4		2.7	
ubiquitin-conjugating enzyme E2	3.3E-72	catalytic domain of Ubiquitin-conjugating enzyme E2	2.5		1.9	
laccase-like protein	3.2E-43	—	—	−1.5		−2.4

Immune signal transduction is an important process supervising an appropriate response against microorganisms. The macrophage migration inhibitory factor (MIF) was found two times more abundant in the plasma of *D. polymorpha* exposed to both *C. parvum* and RNA poly I:C in comparison with the non-exposed hemolymph (Table 2). MIF is a pro-inflammatory cytokine that plays a major role in the regulation of innate immunity against bacteria, protozoa and viruses^40^. Several studies reported the involvement of such proteins in the immune defence of bivalves. MIF cytokines from hemocytes have been found modulated under bacterial challenge in several bivalves such as the scallop *Chlamys farreri*, the clam *Ruditapes philippinarium*, the mussel *Mytilus galloprovincialis* and the pearl oyster *Pinctada fucata*^41–43^. These studies concluded that MIFs are involved in proinflammatory responses and promote hemocytes migration to the wound healing site. To our knowledge, it is the first report of a modulation of MIF in the hemolymph of bivalves exposed to a protozoan and RNA poly I:C. These results suggest that both *C. parvum* and RNA poly I:C may induce pro-inflammatory responses in *D. polymorpha* hemolymph.

The GSEA revealed that locomotion, and cytoskeleton organization BPs were enriched in the hemolymph exposed to protozoa and dsRNA (Table 1). Such result may indicate an activation of hemocytes to counteract *C. parvum* and RNA poly I:C. Regarding the modulated proteins, integrins and myosin-2 were significantly upregulated in hemocytes and plasma in both exposure conditions (Table 2). Integrins are transmembrane receptors involved in adhesion and internalization of microorganisms^44^. Their role in the immune defence of invertebrates have been evidenced in numerous studies^19,45–47^. These authors showed that integrins are involved in the immune defence against bacteria, viruses and protozoa, by promoting cytoskeleton remodeling, locomotion, cell-adhesion, phagocytosis and participate to immune signaling of hemocytes. Myosin is a protein that converts ATP molecules to mechanical energy, generating physical force and movement^48^. While myosin proteins are famous for their involvement in muscle contraction, they are also involved in a variety of movements of non-muscle cells. In hemocytes, myosin-2 is the major motor of cytoskeletal contraction for generating cell movement^49^. In particular, myosin-2 is responsible for many types of cell movements, generating forces behind cell motility and phagocytosis^49,50^. Overall, the enriched BPs and the modulation of both integrins and myosin-2 in *D. polymorpha* hemolymph exposed to *C. parvum* and RNA poly I:C suggest an interaction between hemocytes and microorganisms. Recruitment and migration of hemocytes to the site of infection is one of the key steps to neutralize invaders^51^. Cell motility also contribute to the phagocytosis of invading microbes and encapsulation of larger or refractive invaders. In *D. polymorpha*, interaction between hemocytes and protozoa have already been evidenced by Le Guernic *et al*.^10^. The authors observed hemocyte aggregates around the protozoa *Toxoplasma gondii* and *Giardia duodenalis*, suggesting encapsulation processes to neutralize them. Moreover, Palos-Ladeiro *et al*.^9^ have evidenced a phagocytosis of *C. parvum* oocysts by zebra hemocytes during *ex vivo* challenges.

Antimicrobial peptides (AMP), hydrolases, proteases and protease inhibitors are known to play a crucial role in the elimination of microorganisms by animals. Some proteins considered as AMP were induced in both *C. parvum* and RNA poly I:C exposures (Table 2). Among them, histone H2A was upregulated in hemocytes with a fold change higher than 4.5. The role of histones in the immune responses of aquatic invertebrates was reviewed by Nikapitiya^52^. Histones H4, H2A and H2B of aquatic invertebrates were up-regulated in response to a variety of environmental stressors, including microbes such as viruses and protozoa. These proteins exert antimicrobial activity against bacteria, fungi, viruses, protozoa and recent studies showed that histones are involved in DNA extracellular trap mechanisms that neutralize invaders^53,54^. In addition to histones, several proteolytic and hydrolytic proteins were modulated in both exposure conditions. In the plasma from both exposure conditions, a papain-like cysteine protease was the most up-regulated protease with a fold change greater than 4.4. A trypsin-like serine protease was also up-regulated with a fold change greater than 2.9 while a metalloproteinase-like protein was down-regulated. Proteases, protease inhibitors and hydrolytic enzymes are considered as powerful weapons against microbes in bivalve immunity^5^. Indeed, the concerted action of proteases and inhibitors is involved in several immune processes by cleaving regulator subunits of endogenous proteins, leading to their biological activation and/or exogenous proteins produced by microbes and parasites, leading to their inactivation and/or destruction^23^. Among them, cysteine proteases and their inhibitors such as cystatins play a central role in the Host-Pathogen Cross Talk^55^. A sucrase-isomaltase, was also up-regulated in the plasma challenged with *C. parvum* (FC: 1.4) and RNA poly I:C (FC: 2.2). This digestive-enzyme possess a glycoside hydrolase domain, which may contribute to the destruction of protozoa and viruses by hydrolysis of their glycosidic bonds in *D. polymorpha* plasma.

While biological process related to the complement system were exclusively enriched in the hemolymph exposed to RNA poly I:C (Table 1), many proteins related to the complement system were modulated in both *C. parvum* and RNA poly I:C challenges (Table 2). Among them, complement C2/factor B-like and several C3 complement components were significantly up-regulated or down-regulated in the plasma of *D. polymorpha* exposed to *C. parvum* and RNA poly I:C. The complement system is a sophisticated proteolytic system which acts as a critical first-line defence of innate immunity by promoting the recognition, phagocytosis, elimination of microorganisms^56^. While the existence of the complement system in bivalves remains contradicted, an increasing number of studies described the presence of a proto-complement system in the hemolymph of bivalves, including mussels, oysters and razor clam^23,57–59^. Several complement-related proteins (i.e. C3 component, complement factor-B) of bivalves have been shown to interact with several microorganisms such as bacteria, viruses and protozoa^58,60–62^. The C3 component plays a pivotal role in the complement system and was considered as the “Swiss Army Knife” of cell defence by Ricklin *et al*.^63^. Complement factor-B is a proteolytic enzyme which may act as a regulator of bivalve complement system by cleaving C3 components^60^. In *D. polymorpha* hemolymph, C3 complement components are the major proteins of plasma^15^ and both exposure to *C. parvum* and RNA poly I:C resulted in the modulation of the abundances of complement-related proteins. Taking together, these results suggest a strong involvement of the complement system in the immune defence occurring in the hemolymph of *D. polymorpha*.

#### Immune proteins modulated exclusively in *C. parvum* exposure

The biological interactions between protozoa and zebra mussel have only been studied at the cellular level. Studies have shown that protozoa bioaccumulated by the mussel interact with the immune system of *D. polymorpha* by inducing changes in apoptosis and/or phagocytosis capacity of hemocytes^9–11^. The comparative analysis conducted in hemocytes and plasma exposed *to C. parvum* led to the identification of numerous immune-related proteins involved exclusively modulated in *C. parvum* exposure (Table 3).

**Table 3:** Immune-related proteins significantly modulated exclusively in the hemolymph of *D. polymorpha* challenged with *C. parvum*. The BLAST annotation and expect value are derived from the sequence similarity search against NCBInr database. H = hemocytes and P = plasma and the fold change of significantly modulated proteins (p-value < 0.05) between treated and control sample is indicated.

BLAST annotation (NCBInr)			*C. parvum*	
Immune proteins	E-value	Conserved domain	H	P
**Pattern recognition receptors (PRRs)**				
galectin-9 like	2.0E-32	Galactoside-binding lectin domain	1.6	—
fibrinogen-like protein	2.2E-28	Fibrinogen-related domains	—	2.3
**Immune signaling**				
Signal transducer and activator of transcription 5B (STAT)	1.9E-38	STAT domain, DNA binding domain	−2.6	—
14-3-3 protein beta/alpha	1.6E-89	14-3-3 protein domain	1.5	—
Tumor necrosis factor alpha-induced protein	4.9E-77	Domain of unknown function	1.9	—
**Vesicle trafficking**				
Ras-related protein Rab-10-like	4.5E-91	Rab GTPase family	1.3	—
Ras-related protein Rab-14-like	5.2E-115	Rab GTPase family	1.5	—
Ras-related protein Rab-2-like	6.1E-112	Rab GTPase family	1.3	—
**Microorganism Internalization**				
actin-related protein 2/3 complex subunit 3-like	1.7E-80	ARP2/3 complex ARPC3 (21 kDa) subunit;	1.9	1.7
ras-related C3 botulinum toxin substrate 1 (RAC1)	2.9E-43	Ras-related C3 botulinum toxin substrate 1	1.6	—
Cell division control protein 42 (CDC42)	3.6E-96	CDC 42 domain	1.4	—
neural Wiskott-Aldrich syndrome protein	9.0E-20	—	−1.6	—
clathrin heavy chain	0.0E + 00	Clathrin heavy chain repeat domains	−10.1	1.6
**Immune effectors**				
Antimicrobial peptides				
histone H2B	4.5E-54	Core histone H2A/H2B	1.4	—
Ferritin	2.1E-76	Ferritin-like superfamily	1.5	—
Proteases, protease inhibitors and hydrolases				
cathepsin K-like	1.1E-13	Papain family cysteine protease	1.7	—
cathepsin B	4.9E-53	Papain family cysteine protease	1.7	—
cathepsin L1-like	1.3E-20	Papain family cysteine protease	1.7	—
PREDICTED: serine protease 27 isoform X2	6.2E-07	Trypsin-like serine protease	—	2.1
cathepsin D	1.1E-149	Papain family cysteine protease	—	1.5
**Apoptosis**				
Apoptosis regulator BAX-like	3.9E-60	Apoptosis regulator proteins of the Bcl-2 family	1.3	—
caspase 3	5.2E-14	Caspase domain	1.3	—
caspase 8	1.2E-82	Caspase domain, Death effector domain	−2.2	—
cytochrome c	2.8E-40	Cytochrome c specific domain	1.6	—
voltage-dependent anion channel 2-like	4.4E-99	Eukaryotic porin family	1.3	—
**Other process**				
Cu/Zn-superoxide dismutase	2.2E-67	Copper/zinc superoxide dismutase domain	1.5	
manganese superoxide dismutase	1.2E-52	Iron/manganese superoxide dismutases domain	2	
Dual oxidase	0.0E + 00	NADPH oxidase domain, peroxidases domains	−1.9	

Among the PRRs modulated exclusively in the plasma exposed to *C. parvum*, a Fibrinogen-related protein (FReD) was observed twice more abundant compared to control condition, and galectin-like protein was up-regulated in hemocytes (FC: 1.6). In invertebrates, the primary role of FReDs is defence^64^. Molluscan FReDs are highly diversified molecules involved in pathogen recognition^65^. Numerous studies have reported up-regulation of FReD genes in the hemolymph of bivalve challenged with bacteria and protozoa^66–68^. Authors agreed that FReDs are able to recognize and bind carbohydrates in pathogens and activate immune responses such as the complement system^69^. In the hemolymph of *D. polymorpha*, the up regulation of a FReD in plasma exposed to *C. parvum* may indicate that FReDs contribute to the recognition and destruction of protozoa.

Tumor necrosis factor alpha (TNFα; FC: 1.9) induced protein and 14-3-3 epsilon protein (FC :1.5) were significantly up-regulated while a signal transducer and activator of transcription (STAT) was down-regulated in hemocytes. The induction of TNF-α signaling pathway significantly reduces *C. parvum* spreading in vertebrates cells^70^. This signaling pathway regulates several immune processes including apoptosis, inflammation and production of immune effectors during the acute phase of inflammation and infection^71^. TNFα were up-regulated in *Crassostrea gigas* hemocytes when oysters were challenged with lipopolysaccharide, inducing apoptosis and phagocytosis and the regulation of anti-bacterial activity^72^. The high up-regulation of the abundance of TNFα-induced protein in the *D. polymorpha* hemocytes suggests an induction of the TNFα signaling pathway in early response to *C. parvum*. Activation of STAT signaling has been reported previously in protozoa infections, including *C. parvum*^73,74^. In both vertebrates and invertebrates, STAT proteins interact with the Janus kinases (JAK) to modulate various biological cellular processes including apoptosis, growth, immune system, and inflammation^75^. Research on bivalve immunity agrees that the JAK/STAT pathway is the main regulator pathway of the anti-viral response. In other invertebrates such as flies and shrimps, two studies have reported a modulation of STATs during exposure to plasmodium and peptidoglycans^76,77^. While STAT was down regulated in *D. polymorpha* hemocytes exposed to *C. parvum*, no modulation was observed for the RNA poly I:C challenge. STAT proteins are certainly involved in the immune response of *D. polymorpha* but further analysis is needed to clarify their role in the interaction with microorganisms.

Vesicle transport and Ras protein signal transduction BPs were enriched in the hemolymph exposed to *C. parvum* (Table 1). In addition, several Ras-related proteins of the Rab-family were modulated in hemocytes (Table 3). In both vertebrates and invertebrates, Rab-like proteins are considered as important regulators of intracellular transport and fusion of intracellular structures^78^. Vesicle trafficking is crucial for several immune processes. For example, the release of immune proteins in plasma and the endocytosis of microorganisms necessarily involves a significant vesicular traffic and fusion^79^. Biological processes related to the internalization of microorganisms were also enriched in the hemolymph exposed to *C. parvum*, including actin polymerization or depolymerization, ARP2/3 complex-mediated actin nucleation and lamellipodium assembly BPs (Table 1). In addition, numerous proteins related to the actin-related protein 2/3 complex (Arp 2/3) were modulated exclusively in the hemocytes challenged with *C. parvum* (Table 3). Among them actin-related protein 2/3 complex subunit (FC: 1.9), ras-related C3 botulinum toxin substrate (FC: 1.6), Cell division control protein 42 (FC: 1.4) were upregulated while the neural Wiskott-Aldrich syndrome protein was down regulated (FC: −1.6). The Arp 2/3 complex is a membrane complex that is considered to play a central role in the assembly of actin filaments during the migration and phagocytosis processes of macrophages^80,81^. In human cells, implication of the Arp 2/3 complex during *C. parvum* infection has been underlined by Elliott *et al*.^82^. The authors showed that *C. parvum* induced actin polymerization at sites of infection by triggering nucleation machinery of the Arp 2/3 complex. Focusing on hemocytes, Lau *et al*.^83^ observed an up-regulation of Arp 2/3-related genes correlated with an increase of cell motility when hemocytes of the oyster *Crassostrea virgnica* were *in vitro* challenged with the intracellular parasite *Perkinsus marinus*. In shrimp exposed to the bacteria *Vibrio anguillarum*, Xu *et al*.^84^ observed a modulation of genes involved in the regulation of Arp 2/3 complex when bacteria were phagocyted by hemocytes. Studies have observed, by flow cytometry analyses, a phagocytosis of *C. parvum* oocysts by zebra mussel hemocytes after 4 hours of *ex vivo* exposure^9,85^. As evidenced by both BP enrichment analysis and BLAST annotation of modulated proteins, our results may indicate that *C. parvum* are phagocyted by *D. polymorpha via* the Arp 2/3 complex.

Proteins devoted to the destruction of *C. parvum* were mainly modulated in the hemocytes (Table 2). Numerous cathepsins were upregulated in hemocytes exposed to *C. parvum*. Of them three different types of cathepsins were up-regulated in hemocytes with a fold change higher than 1.7 and a cathepsin D was up-regulated in plasma. Cathepsins have been subject to several investigation and are linked to the immune defence in bivalves^23^. Cathepsins are lysosomal cysteine proteases that regulate the immune and cell death process and contribute to the degradation of phagocyted microorganisms^86^. The high number of modulated cathepsins in *D. polymorpha* hemocytes strongly suggest that the lysosome-mediated antimicrobial defence is engaged against *C. parvum*. Histone H2B (FC: 1.4) and Ferritin (FC: 1.5) were also up-regulated exclusively in the hemocytes exposed to C*. parvum*. To survive and replicate in hosts, many protozoan invaders secure host iron^87^. Ferritins are iron-sequestering proteins that regulate iron homeostasis in cells and tissues, which are described as important immune regulators that limit the spreading of microbes such as bacteria^88^. Numerous studies highlighted the immune function of ferritins in bivalve. In scallop and freshwater mussel, ferritin genes were up-regulated in response to bacteria correlated with an inhibition of bacteria growth^88–90^. Furthermore, in response to quahog parasite unknown (QPX), ferritin genes were highly expressed in mantle and gill tissues of the clam *Mercenaria mercenaria*^91^. Iron is probably an important element for the proliferation of *C. parvum*. The up regulation of the abundance of a ferritin in the hemocytes exposed to *C. parvum* may suggest that this protein prevents the growth of *C. parvum* in zebra mussel hemolymph.

The modulation of apoptosis in the hemolymph exposed to *C. parvum* were evidenced by both BPs enrichment analysis and the BLAST annotation of modulated proteins. Indeed, the regulation of apoptotic signaling pathway and apoptotic cell clearance BPs were significantly enriched in the hemolymph challenged with *C. parvum*. Key proteins involved in the regulation and induction of the intrinsic apoptotic pathway were modulated in hemocytes. Among them, the apoptosis regulator BAX, caspases, cytochrome C and voltage-dependent anion channel were up-regulated in hemocytes challenged with *C. parvum* while another caspase was down regulated. Apoptosis is fundamental biological process involved in immune system homeostasis by preventing inflammatory damage and limiting the spread of pathogens^92^. While apoptosis is described as a mechanism that removes parasite cells, it has been shown induction of inhibition of programmed cell death by protozoa can be linked to parasite survival strategies^93^. In bivalve hemocytes, apoptosis is a common response to protozoa^94,95^. In particular, the involvement of apoptosis in interactions between zebra mussel hemocytes and their intracellular parasite has been evidenced by Minguez *et al*.^11^.

Finally, other proteins exclusively modulated in the hemolymph exposed to *C. parvum* were related to oxidative metabolism. Of them, a dual oxidase (Duox) was down-regulated in hemocytes. Duox proteins are well-defined sources of reactive oxygen species (ROS) production^96^. ROS production in bivalve hemocytes is regarded a conserved and widespread immune defence mechanisms in response to microorganisms, since cytotoxic ROS interact with a wide variety of organic and inorganic molecules, altering their functions^97,98^. Induction of Duox genes have been associated to successful defence response against microorganisms in several bivalves, probably by promoting the production of ROS that degrade internalized microorganisms^99,100^. In this context, the modulation of a Duox protein in hemocytes exposed to *C. parvum* may indicate that ROS-dependent killing processes are involved in defence against *C. parvum*. While ROS have a biocidal effect on invading microbes, ROS production can also injure the cells of the host by causing oxidative stress. In this case, the excess ROS should be removed by antioxidant enzymes to avoid damage on host cells. Noteworthy, two antioxidant enzymes were upregulated in *D. polymorpha* hemocytes exposed to *C. parvum*. More specifically, a manganese superoxide dismutase (SOD) (FC: 2.0) and CU/ZN SOD (FC: 1.5) were up regulated in hemocytes. SOD are anti-oxydant enzymes involved in antioxidative defence system, by protecting organisms from excess of ROS and maintaining the redox balance of immune system^101^. In the oyster *C. gigas*, SODs considered as the major protein group in plasma was able to bind to various microorganisms^102^. In addition to its antioxidant activity, extracellular SODs enhanced cellular immune response in oysters exposed to *Vibrio splendidus* by acting as a PRR and by contributing to the immune priming of oysters^103^.

#### Immune proteins modulated exclusively in RNA poly I:C exposure

Molecular mechanisms involved in anti-viral responses of bivalves remained till now the “blackbox” to solve since tractable systems to induce viral infection have only recently developed^5^. Recently, the double-stranded RNA poly I:C emerged as a powerful inducer of antiviral response in bivalve species^16,104^. In this study, the hemolymph of *D. polymorpha* was challenged with RNA poly I:C to simulate a viral infection and the proteins involved in the antiviral defence of zebra mussels were identified. Our results have shown that the most enriched BPs related to immunity were “response to stress” and “response to chemical”, both representing more than 27% of the modulated proteins in the hemolymph challenged with RNA poly I:C (Table 1). The GSEA also revealed that immune system processes were modulated in the hemolymph exposed to RNA poly I:C since many BPs related to immunity were enriched, including the immune system process and humoral immune response BPs. Recent results suggest that antiviral defence of bivalves are executed through an interferon pathway like in vertebrates. Key proteins involved in the interferon pathway were identified in the hemolymph of *D. polymorpha*^15^. In our study, no proteins related to the interferon pathway were detected as modulated in response to RNA poly I:C. However, many proteins that have already shown interactions with viruses in non-bivalve organisms were modulated exclusively in RNA poly I:C exposure, suggesting a potential role in the anti-viral responses of *D. polymorpha*. In contrast with the hemolymph exposed to *C. parvum*, immune proteins exclusively modulated in the hemolymph challenged with RNA poly I:C were mainly modulated in the plasma fraction (Table 4). Like in other marine bivalves, this result suggests that plasma proteins may play a central role in *D. polymorpha* antiviral response^17^.

**Table 4:** Immune related proteins modulated exclusively in the hemolymph of *D. polymorpha* challenged with RNA poly I:C. The BLAST annotation and expect value are derived from the sequence similarity search against NCBInr database. H= hemocytes and P= plasma and the fold change of significantly modulated proteins (p-value < 0.05) between treated and control sample is indicated.

BLAST annotation (NCBInr)			RNA poly I:C	
Immune proteins	E-value	Conserved domain	H	P
**Pattern recognition receptors (PRRs)**				
Peptidoglycan-recognition protein	1.9E-40	Peptidoglycan recognition proteins, lysozyme domain	—	−1.5
Beta-galactoside-binding lectin	2.0E-13	Galactoside-binding lectin	—	−1.5
**Immune signaling**				
Granulin	1.0E-28	Granulin domain	—	1.7
**cell-adhesion**				
Cadherin-23 (Otocadherin)	1.2E-205	Cadherin repeat-like domains	—	3.2
cadherin-like protein	0.0E + 00	Cadherin repeat-like domains	—	2.9
fibronectin-like protein	4.0E-59	Fibronectin type III domain, Immunoglobulin domain	—	2
**Immune effectors**				
Proteases, protease inhibitors and hydrolases				
Cathepsin K	5.4E-28	Papain family cysteine protease	−1.3	—
Alpha-2-macroglobulin	2.7E-07		—	1.6
Serpin B6	1.3E-38	SERine Proteinase INhibitors	—	−1.8
Lysosomal alpha-mannosidase	0.0E + 00	Glycosyl hydrolases family 38 C-terminal domain, Alpha mannosidase, N-terminal catalytic domain ofcore-specific lysosomal alpha 1,6-mannosidas	—	−1.4
Antiviral proteins?				
Ectonucleotide pyrophosphatase /phosphodiesterase	0.0E + 00	Ectonucleotide pyrophosphatase/phosphodiesterase, DNA/RNA non-specific endonuclease	—	4.6
Spliceosome RNA helicase	7.4E-31	DEAD box helicase domain	−1.5	-
Thaumatin-like protein	3.7E-83	antifungal thaumatin-like domain	—	−1.5
Mucin-like protein	7.5E-56	Anthrax Toxin Receptor, Herpes virus major outer envelope glycoprotein (BLLF1), Endomucin domain	—	2.2
Complement system				
complement factor B-like protein	1.2E-04	Trypsin-like serine protease	—	2.7
Complement C1q tumor necrosis factor	3.8E-04	C1q domain	—	−1.4

Of the proteins acting as PRRs, a Peptidoglycan-recognition protein (PGPR) containing a lysozyme domain and a galectin protein were exclusively modulated in the plasma exposed to RNA poly I:C (Table 4). PGRPs have multiple immune-related functions^105^. PGRPs were first considered as a PRRs that recognize peptidoglycans from bacteria cell walls^106^. In addition to their role of recognition of microbes, a lysozyme domain was observed in the PGRP of oyster hemocytes which may confer a lytic function^107^. In insects PRPGs interact with several immune signaling pathways^108^. Involvement of PRPG in the immune defence against bacterial invasions was evidenced in several bivalve species such as the manila clam *Ruditapes philippinarum*, the bay scallop and the pacific oyster^107,109,110^. Few studies have investigated the role of PRPGs in anti-viral defence. In the grass carp and human cells, PRPGs genes were strongly expressed during infection with dsRNA poly I:C^111,112^. In the silkworms *Bombyx mori*, PRPGs are believe to exert distinct functions in host responses to bacteria and viruses^105^. Authors suggest that silkworms PRPG act as PRR in response to bacteria while PRPGs regulate immune signaling during viral infection. Recently, Wang *et al*.^113^ observed an up-regulation of PRPGs in the oyster *C. gigas* challenged with RNA poly I:C and considered PRPGs as a PRRs involved in virus recognition.

A granulin protein was up-regulated in plasma exposed to RNA poly I:C (FC:1.7). In vertebrates, granulins are considered as important modulators of cell growth and are believed to be involved in the immune signaling^114^. Furthermore granulins were able to bind synthetic DNA molecules and were considered as a central actor in the signaling of viral invasion^115^. Especially, granulins were described as soluble co-factor of the toll-like receptor signaling pathway in macrophages^116^. To our knowledge, the role of granulins in the immune system of invertebrates has only been studied in a context of snail infection by trematodes^117,118^. In these studies, granulins are described as a cytokine that promotes hemocyte activation and proliferation, making the snail resistant to its parasite. The up-regulation of the extracellular granulin-like protein in the hemolymph of *D. polymorpha* challenged with RNA poly I:C suggest that granulins participate to the immune signaling against dsRNA.

Proteins involved in nucleotide metabolism were also modulated in response to RNA poly I:C. An RNA helicase protein containing a DEAD box helicase domain was down-regulated in hemocytes while an ectonucleotide pyrophosphatase/phosphodiesterase (ENPP) protein containing an DNA/RNA nuclease domain was up-regulated in plasma with a fold change greater than 4.5. The role of RNA helicases in anti-viral responses has been subject to extensive investigations and contribute as both sensors and effectors of antiviral immunity^119^. RNA helicases have been shown to be up regulated in the hemocyte of shrimp infected by the WSSV^120^. In bivalves, retinoic acid-inducible gene-I-like receptors (RIG-like receptors) which contain a DEAD box RNA helicase are recognized as fundamental receptors of viral infections^23^. The RNA helicase modulated in the hemolymph of *D. polymorpha* exposed to RNA poly I:C is probably a protein that functions similarly to RIG-like proteins found in bivalves. ENPPs hydrolyze phosphates from nucleotides and their derivatives, and the extracellular form have already been linked to viral infections. For example, ENPP was one of the most significantly up-regulated secretory proteins associated with *Hepatitis* B virus replication in human cells^121^. The ENPP modulated in *D. polymorpha* contained a DNA/RNA non-specific nuclease domain whose function is to cleave double-stranded and single-stranded nucleic acids^122^. In insects, *ex vivo* assay revealed that dsRNA was rapidly degraded in the plasma by proteins that contain a DNA/RNA nuclease domain. The conserved domains of both RNA helicase and ENPP and their function in nucleic acid biology suggest their involvement in *D. polymorpha* response to dsRNA.

The abundance of a Thaumatin-like protein (TLP) was significantly down-regulated in the plasma of exposed to RNA poly I:C (FC: −1.5) (Table 4). In plants, TLPs are believe to exert antifungal activity, xylanase inhibitor activity and glucan binding and glucanase activities^123^. In bivalves, information on TLP functions are relatively scarce. To our knowledge, only Niu *et al*.^124^ investigated the role of TLPs in the oyster *C. gigas*. Authors observed a higher expression level of TLPs genes in immune-related organs such as hemocytes and hepatopancreas. Moreover, they observed an up-regulation of the expression of TLP genes in oysters challenged with mannan, *Pichia pastoris* yeast and RNA poly I:C. They concluded that TLPs play a vital role in defending against fungal infection. In our study, TLPs were modulated in the plasma of *D. polymorpha* exposed to RNA poly I:C suggest their involvement in zebra mussel immunity. A mucin-like protein was also significantly modulated in the plasma exposed to RNA poly I:C with a fold change greater than 2.Lieleg *et al*.^125^ considered that mucin biopolymers exert a broad-spectrum antiviral activity. Thus, it is not excluded that this mucin-like protein act as anti-viral agents of the *D. polymorpha* hemolymph.

Other immune proteins exclusively modulated by RNA poly I:C were related to proteolytic and hydrolytic enzymes. Among them, the protease inhibitor alpha-2 macroglobulin was modulated with a fold change greater than 1.5 in plasma. Serine-protease inhibitor (Serpin) and lysosomal alpha-mannosidase were down-regulated in the plasma exposed to RNA poly I:C.

### Towards the identification of new immunomarkers for ecotoxicological studies

**Figure 3:**
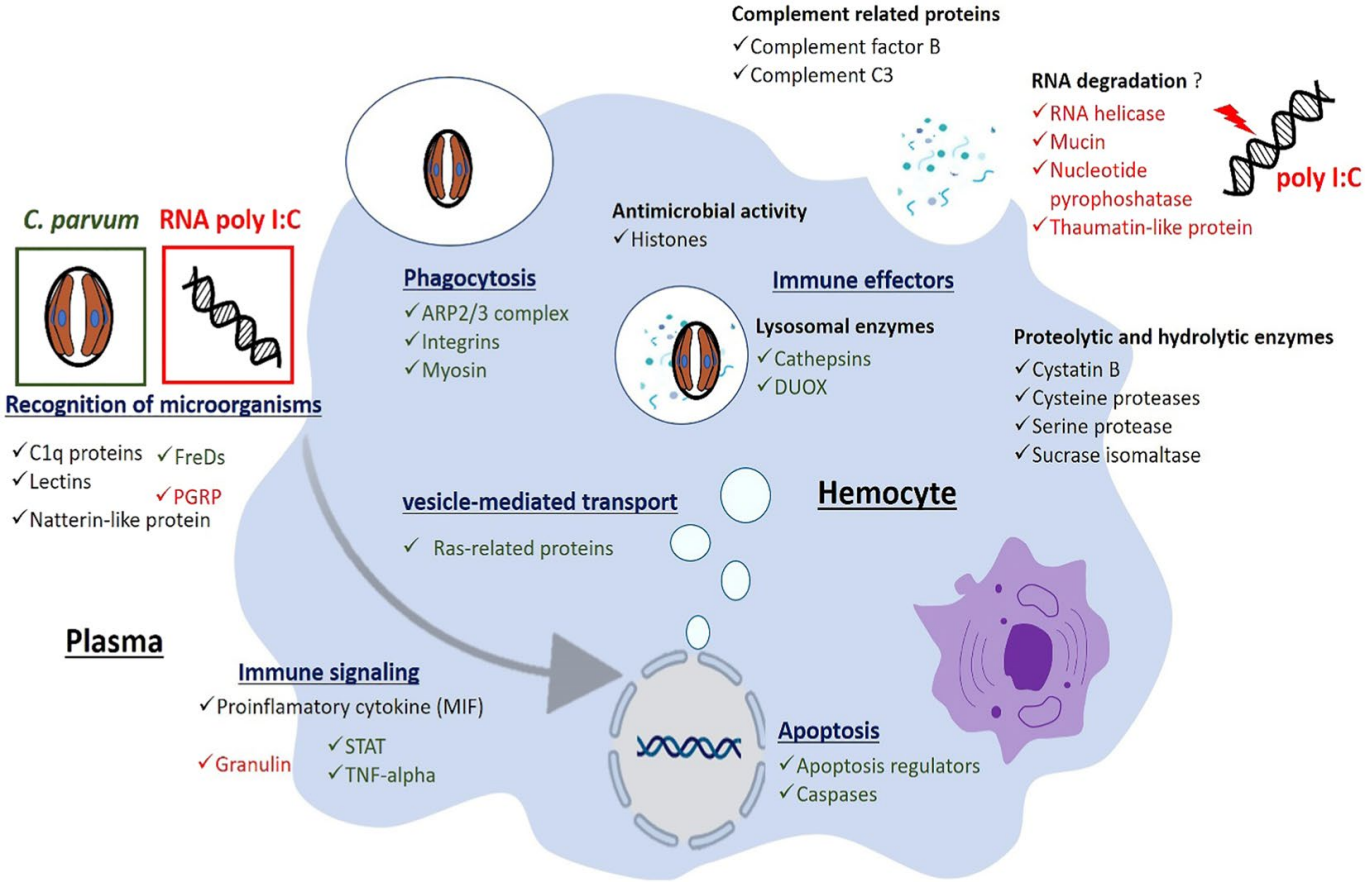
Summary of immune proteins from *D. polymorpha* hemolymph modulated during interaction with microorganisms. Proteins written in black were modulated by both *C. parvum* and RNA poly I:C, the green ones were exclusively modulated in *C. parvum* exposure while the red ones were exclusively modulated in RNA poly I:C exposure.

The zebra mussel, *Dreissena polymorpha* is a sentinel species used in aquatic ecotoxicology in part because this filter bivalve accumulates and concentrates a large number of biological and chemical contaminants^1^. Current studies seek to understand the interaction between contaminations and biological responses of *D. polymorpha* in order to assess the impacts of contaminants on living organisms and ecosystems. Among biological processes, the immune system contributes to ensuring the integrity of the host organism by eliminating foreign constituents such as microorganisms. Investigations have shown that many molecules released in the environment are likely to alter the immune system of organisms, thus posing a risk to the health of individuals and populations. Numerous studies have shown that the immune system of bivalves is sensitive to a broad range of chemical contaminants which affect their immunocompetence^126^. The comparative proteomic analysis performed on *D. polymorpha* hemolymph exposed to *C. parvum* and RNA poly I:C provided the identification of numerous proteins reflecting a large panel of immune processes (Fig. 3). In both exposure conditions, several proteins involved in cell motility and destruction of microorganisms were modulated. In *C. parvum* challenge, the comparative proteomic analysis resulted in the identification of key proteins involved in immune signaling, phagocytosis and apoptosis process as well as proteins of the lysosome-mediated antimicrobial defence. Moreover proteins that may act in anti-viral defence were observed in the hemolymph exposed to RNA poly I:C. All these proteins constitute potential immunomarkers for evaluating the impact of contaminants on zebra mussel immunity.

In this study, the hemolymph was challenged during 4 h to *C. parvum* and RNA poly I:C in *ex vivo* conditions. *Ex vivo* exposures were initially designed to simplify the biological system and promote a strong interaction between hemocytes and biological stressors. This strategy has proven its efficiency for identifying proteins that may play a key role in the management of *C. parvum* and RNA poly I:C. However, such exposures do not take into account the integrity of the body’s biology and the dynamic of microorganisms within mussel tissues. We recommend that future exposure should be performed with longer *in vivo* condition in order to be as close as possible to biological reality. *In vivo* assays are more appropriate for studying the immune response of organisms because changes in immune responses may be related to hormonal, nutritional, physical and biological alterations such as changes in the microbiota^127–129^. Furthermore, our results only give a picture of the early immune responses occurring after 4 h of exposure. Experimental challenges with different kinetics of contamination would provide a better understanding of the involvement of immune proteins in the contaminant management. Finally, the present differential proteomics performed with the hemolymph of *D. polymorpha* has led to the identification of key proteins reflecting a variety of immune mechanisms, including recognition, internalization and destruction of microorganisms. In addition to providing information on host-microbial interaction in the zebra mussel, this study paves the way for the identification of new immunomarkers that reflect the broad immune processes of *D. polymorpha*. The modulation of these proteins and the analysis of their genetic regulation should be further validated by other methodologies, such as targeted proteomics and RNAseq.^14,130^. Once approved, these immunomarkers will provide a better understanding of the impact of biological contaminations on the immune system of aquatic organisms. Finally, work remains for a better understanding of the immune response of *D. polymorpha*. For example our results should be in the future further complemented with studies regarding immune signaling pathways, and could be compared with results obtained in other invertebrates, such as bivalves or insects^131,132^.

## Conclusion

Overall, this study helped to understand the interaction between the immune system of *D. polymorpha* and microorganisms. The comparative proteomic analysis performed on hemocytes and plasma exposed to *C. parvum* and RNA poly I:C has provided a large set of information on the immune processes involved in anti-protozoal and anti-viral defence of *D. polymorpha*. The GSEA and the BLAST annotation performed on modulated proteins have shown that common immune responses were induced by *C. parvum* and RNA poly I:C. Indeed, several proteins involved in the recognition and destruction were modulated in both exposure conditions. Among them, proteins related to the complement system were up and down regulated, suggesting a significant implication of the complement system in *D. polymorpha* immune defence. Other immune-related proteins were exclusively modulated in *C. parvum* or RNA poly I:C exposures, which suggest a certain immune specificity depending on the nature of encountered microbes. Multiple clues showed that *C. parvum* was managed by hemocytes of *D. polymorpha* hemolymph. Firstly, a higher number of proteins were modulated in hemocytes compared to plasma. Then, key proteins involved in phagocytosis and apoptosis process as well as proteins of the lysosome-mediated antimicrobial defence were modulated in hemocytes exposed to *C. parvum*. In contrast, a higher number of modulated proteins was observed in plasma for the RNA poly I:C challenge, which suggests that at four hours of exposure dsRNA is managed in the extracellular medium of hemolymph. Among the proteins modulated exclusively in the RNA poly I:C challenge, potential antiviral effectors were identified. This study provides a better understanding on interaction between zebra mussels and microbes. It also contributes to the characterization of the molecular mechanisms involved in the immune defence of zebra mussels and paves the way to the identification of new immunomarkers to assess the impact of contaminants on aquatic organisms and ecosystems.

## Material and Methods

### Sampling and maintenance of animals

Sampling and maintenance of animals were performed as described in Leprêtre *et al*.^15^. Zebra mussels between 15- and 25-mm long were collected at the Der lake (Grand Est, France) in May 2017. Mussels were maintained for at least two weeks at the laboratory in 20 L tanks filled with spring water Cristaline Aurèle (Jandun, France), at constant temperature (14 °C) and with a controlled aeration. Mussels were fed twice a week with a mixture of two species of microalgae, *Chlorella pyrenoidosa* and *Scenedesmus obliquus* (two million of microalgae per day and per mussel). Tank water was removed weekly.

### *Ex vivo* challenge experiment

After two weeks of acclimation in the laboratory, 150 µL of hemolymph per mussel was withdrawn from the posterior adductor muscle by gentle aspiration with a 30 g × 0.3 mL syringe. The quality of each sample was checked under a microscope for confirming the exclusive presence of hemocytes. Samples were pooled to obtain 3 mL hemolymph. As described in Fig. 1, from this pool (3 mL), 1 mL of hemolymph was distributed in order to perform three exposure conditions: (i) the control condition with hemolymph free, (ii) the anti-protozoal challenge where the hemolymph was exposed to *Cryptosporidium parvum* oocysts purified in phosphate-buffered saline (pH 7.4) (5:1 protozoa:hemocyte; INRA, Val de Loire research center, France) as referred in Le Guernic *et al*.^10^ and (iii) the antiviral challenge where the hemolymph was exposed to 100 μg/ml of RNA poly I:C (Sigma-Aldrich). For all conditions, samples were incubated at 16 °C during 4 hours and the experiment was repeated 5 times in order to obtain 5 replicates per exposure conditions.

### Protein extraction and trypsin digestion

Sample preparation for mass spectrometry analysis was performed as described in Leprêtre *et al*.^15^. Briefly, pools of hemolymph were centrifuged at 800 × *g* for 15 min at 4 °C to separate hemocytes from plasma. 500 µL of Tri-reagent (Sigma-Aldrich) were added to hemocyte pellets and stored at −20 °C until the protein extraction, 3 volumes of ice-cold acetone were immediately added to the plasma and the resulting samples were stored overnight at −20 °C for protein precipitation.

For hemocytes, cells were lysed by passing them ten times through a syringe equipped with a 25 G needle and then centrifuged at 12,000 × *g* during 2 min for an initial clean up. RNAs were removed from the resulting supernatant by adding 100 μL of chloroform, incubating for 15 min, centrifugation at 12,000 ×*g* at 4 °C for 15 min, and removing the colorless aqueous phase. DNA was discarded after adding 150 μL of absolute ethanol for 3 min followed by centrifugation at 2,000 ×*g* at 4 °C for 5 min. Then, proteins from the resulting supernatant were precipitated by adding 3 volumes of ice-cold acetone to the supernatant. Samples were incubated at room temperature for 10 min and then centrifuged during 10 min at 12,000 ×*g* and 4 °C. The remaining protein pellet was then washed three times during 10 min in 1 mL of 0.3 M guanidine hydrochloride in 95% ethanol (Vol:Vol) followed by centrifugation at 8,000 ×*g* and 4 °C for 5 min and removal of the supernatant. A final wash was performed with 1 mL of 95% ethanol before protein pellets were dried at room temperature and re-suspended in 80 μL of resuspension buffer.

Proteins precipitated from plasma were centrifuged at 15,000 ×*g* for 10 min at 4 °C. The protein pellets were washed twice with 1 mL of 80% cold acetone followed by centrifugation at 15,000 ×*g* for 10 min at 4 °C, and removal of the supernatant. Then, the pellets were dried at room temperature and re-suspended in 80 µl of re-suspension buffer consisting of 6 M urea; 2 M thiourea; 4% 3-[(3-cholamidopropyl)dimethyl-ammonio]-1-propanesulfonate (CHAPS); 65 mM dithiothreitol (DTT), 0.1% Triton X100 (Vol:Vol), pH 7.8.

The concentration of total proteins in each sample was measured by Bradford against a bovine serum albumin (BSA) calibration curve. Biological replicates were diluted in re-suspension buffer to obtain the same protein concentration of 2.25 µg/µL. A volume of 20 µL of each sample was diluted with 10 µL of LDS 3X buffer (Invitrogen) consisting of 80 mM Tris / HCl, 106 mM Tris base, 1.5% lithium dodecyl sulfate, 7.5% glycerol, 0.38 mM EDTA, 0.17 mM SERVA Blue G250, 0.131 mM phenol red, buffered at pH 8.5 and supplemented with 2.5% beta-mercaptoethanol. Then, samples were boiled for 5 min at 95 °C, centrifuged briefly, and loaded into a 4–12% gradient 10-well NuPAGE gel (Invitrogen). A 5 min electrophoretic migration of the proteins was performed at 200 V in MES buffer (Invitrogen). After Coomassie Blue Safe staining (Invitrogen) and destaining with ultra-pure water, each polyacrylamide band corresponding to each whole proteome was excised. Proteins from each polyacrylamide band was first alkylated with iodoacetamide, reduced with DTT and then proteolyzed with Sequencing Grade Trypsin (Roche) in presence of 0.01% ProteaseMAX surfactant (Promega) for 1 h at 50 °C as previously described^133^. The proteolysis was then stopped with the addition of TFA 5% to reach a final concentration of 0.5%.

### NanoLC-MS/MS analysis

The resulting peptide mixtures were analyzed in a data-dependent mode with a Q Exactive HF tandem mass spectrometer (ThermoFischer Scientific) coupled on line to an Ultimate 3000 chromatography system (ThermoFisher), essentially as previously described^134^. A volume of 5 µL of each sample was injected, first desalted with a reverse-phase capillary precolumn C18 PepMap 100 (LC-Packing) and then resolved with a nanoscale 500 mm C18 PepMap 100 (LC-Packing) column using a 90 min gradient from 2% to 20% in 70 min and from 20% to 36% in 20 min of acetonitrile, 0.1% formic acid and then 80% for 1 min. Mass spectra of peptide ions with 2+ and 3+ charge states were acquired in the full scan range 350–1800 *m/z*, with a resolution set at 60,000, an AGC target at 3 × 10^6^. MS/MS mass spectra were acquired following a Top20 method for selecting peptides for fragmentation with an AGC target set at 10^5^, a loop count of 60 msec, an isolation window of 1.6 *m/z*, a resolution at 15,000, an exclusion time of 10 sec, and a scan carried out in the mass range from 200 to 2,000 *m/z*.

### Interpretation of MS/MS spectra

MS/MS spectra were assigned by using Mascot search engine against the polypeptide database “Dreissene_cascade”, a database derived from proteogenomic investigations which comprises 54,097 specific polypeptide sequences, totaling 20,419,358 residues^15^. Peptide assignations were validated with the following parameters: full-trypsin specificity, a maximum of two missed cleavages, a mass tolerance of 5 ppm on the parent ion and 0.02 Da on the MS/MS analysis, and a p-value for peptide validation at 0.05. Modifications taken into account were carboxyamidomethylated cysteine (+57.0215) as static modification and oxidized methionine (+15.9949) and deamidation of asparagine and glutamine (+0.9848) as dynamic modifications. A protein was validated when at least two unique peptide sequences were detected in at least one of the 30 samples used in this study. The false positive rate for protein identification was estimated, through a search with the reverse decoy MASCOT option, as less than 1% using the same parameters. For each condition and for each replicate, the numbers of peptide-to-spectrum assignations observed for the different detected peptides of the same polypeptide were summed (spectral counts). Identified proteins which share the same attributed peptides, transcript annotation and BLAST annotation were used for parsimony and creation of protein families for which the longest polypeptide isoform sequence was selected to be the representative.

### Differential proteomics

The abundance of each protein in hemocytes and plasma from the hemolymph exposed to *C. parvum* or RNA poly I:C were compared to the abundance of proteins from the unexposed hemolymph, using the T-fold module of the Patternlab program for proteomics version 4.0.0.59^135^. The comparative analysis of the expression of proteins in plasma and hemocytes was based on the spectral counts obtained with the five replicates of each exposure condition for each protein. The difference was analyzed using a L-stringency set at 0.6, a minimum of four measurements out of five replicates, and normalization of the dataset upon the total signal. From this analysis, proteins were classified into 4 groups: the first ones correspond to identifications that satisfied both the automatic fold-change trheshold (FC > 1.3) and statistical criterion (p-value < 0.05), the second ones did not meet the fold and p-value criteria, the third ones were filtered out by the L-stringency which needs further experimentation to verify if they are indeed differentially abundant and the fourth ones are not considered as they satisfied the fold criterion but not the p-value. The protein classified in the first category were considered as potential protein signatures of the treatment.

### Bioinformatics analysis

BLAST annotation of identified proteins was performed using the BLASTp module of DIAMOND (version 0.8.22). Sequence similarity search with an E-value threshold set at 1E-03 was carried out without taxonomical restriction against two databases: (i) the National Center for Biotechnology Information (NCBI) database of non-redundant protein sequences (nr) (131,025,392 sequences released 05-09-2017) and (ii) the Swissprot database (465,089 sequences released, 30-09-2016). Then a search of the conserved domain (CD) of proteins with the Batch CD-Search tool of NCBI server was performed to support BLAST annotations^136^.

An enrichment analysis of biological processes (BPs) in the hemolymph exposed to *C. parvum* and RNA poly I:C was performed with the Database for Annotation, Visualization and Integrated Discovery (DAVID) tools for GO annotation functional enrichment analysis. Using Uniprot accessions, a Fisher’ exact test was performed on significantly modulated proteins in hemolymph exposed to *C. parvum* or RNA poly I:C to reveal enriched BPs. The entire set of proteins identified in both hemocytes and plasma samples from all samples was used as background to perform the enrichment analysis. Only enriched BPs related to immune process with a α risk lower than 5% are considered in this study.

### Mass spectrometry and proteomics data

The mass spectrometry and proteomics data were deposited to the ProteomeXchange Consortium via the PRIDE^137^ partner repository with the dataset identifier PXD017747 and 10.6019/PXD017747.

## References

[1] Palos Ladeiro, M. *et al*. Mussel as a Tool to Define Continental Watershed Quality. In Organismal and Molecular Malacology (ed. Ray, S.) (2017).

[2] Gu J-D, Mitchell R (2002). Indigenous microflora and opportunistic pathogens of the freshwater zebra mussel, Dreissena polymorpha. Hydrobiologia.

[3] Palos Ladeiro M, Aubert D, Villena I, Geffard A, Bigot A (2014). Bioaccumulation of human waterborne protozoa by zebra mussel (Dreissena polymorpha): Interest for water biomonitoring. Water Research.

[4] Mezzanotte V (2016). Removal of enteric viruses and Escherichia coli from municipal treated effluent by zebra mussels. Science of The Total Environment.

[5] Allam B, Raftos D (2015). Immune responses to infectious diseases in bivalves. Journal of Invertebrate Pathology.

[6] Allam B, Pales Espinosa E (2016). Bivalve immunity and response to infections: Are we looking at the right place?. Fish & Shellfish Immunology.

[7] Zannella C (2017). Microbial Diseases of Bivalve Mollusks: Infections, Immunology and Antimicrobial Defense. Marine Drugs.

[8] Juhel G, Ramsay RM, Davenport J, O’Halloran J, Culloty SC (2015). Effect of the Microcystin-Producing Cyanobacterium, *Microcystis aeruginosa*, on Immune Functions of the Zebra Mussel *Dreissena polymorpha*. Journal of Shellfish Research.

[9] Palos Ladeiro, M. *et al*. Mollusc Bivalves as Indicators of Contamination of Water Bodies by Protozoan Parasites*. In Reference Module in Earth Systems and Environmental Sciences* (2018).

[10] Le Guernic A (2019). First evidence of cytotoxic effects of human protozoan parasites on zebra mussel (*Dreissena polymorpha*) haemocytes. Environmental Microbiology Reports.

[11] Minguez L, Brulé N, Sohm B, Devin S, Giambérini L (2013). Involvement of Apoptosis in Host-Parasite Interactions in the Zebra Mussel. Plos One.

[12] Afroz, A., Zahur, M., Zeeshan, N. & Komatsu, S. Plant-bacterium interactions analyzed by proteomics. *Front. Plant Sci*. **4** (2013). 10.3389/fpls.2013.00021 PMC3573209 23424014

[13] Jean Beltran, P. M., Federspiel, J. D., Sheng, X. & Cristea, I. M. Proteomics and integrative omic approaches for understanding host–pathogen interactions and infectious diseases. *Mol Syst Biol* **13** (2017). 10.15252/msb.20167062 PMC5371729 28348067

[14] Gouveia, D. *et al*. Ecotoxicoproteomics: A decade of progress in our understanding of anthropogenic impact on the environment. *Journal of Proteomics* (2019). 10.1016/j.jprot.2018.12.001 30529745

[15] Leprêtre M (2019). The immune system of the freshwater zebra mussel, Dreissena polymorpha, decrypted by proteogenomics of hemocytes and plasma compartments. J Proteomics.

[16] Green TJ, Montagnani C (2013). Poly I:C induces a protective antiviral immune response in the Pacific oyster (Crassostrea gigas) against subsequent challenge with Ostreid herpesvirus (OsHV-1 μvar). Fish & Shellfish Immunology.

[17] Green TJ, Raftos D, Speck P, Montagnani C (2015). Antiviral immunity in marine molluscs. Journal of General Virology.

[18] Castellanos-Martínez S, Diz AP, Álvarez-Chaver P, Gestal C (2014). Proteomic characterization of the hemolymph of Octopus vulgaris infected by the protozoan parasite *Aggregata octopiana*. Journal of Proteomics.

[19] Fernández-Boo S, Villalba A, Cao A (2016). Protein expression profiling in haemocytes and plasma of the Manila clam *Ruditapes philippinarum* in response to infection with *Perkinsus olseni*. Journal of Fish Diseases.

[20] Graczyk TK, Fayer R, Cranfield MR, Conn DB (1998). Recovery of Waterborne Cryptosporidium parvum Oocysts by Freshwater Benthic Clams (*Corbicula fluminea*). Appl. Environ. Microbiol.

[21] Medina-Gali R (2019). Plasma proteomic analysis of zebrafish following spring viremia of carp virus infection. Fish & Shellfish Immunology.

[22] Tao M (2019). Quantitative serum proteomics analyses reveal shrimp responses against WSSV infection. Developmental & Comparative Immunology.

[23] Gerdol, M. *et al*. Immunity in Molluscs: Recognition and Effector Mechanisms, with a Focus on Bivalvia. In *Advances in Comparative Immunology* (ed. Cooper, E. L.) 225–341 (2018).

[24] Hille-Rehfeld A (1995). Mannose 6-phosphate receptors in sorting and transport of lysosomal enzymes. Biochimica et Biophysica Acta (BBA) - Reviews on Biomembranes.

[25] Díaz-Salinas MA, Casorla LA, López T, López S, Arias CF (2018). Most rotavirus strains require the cation-independent mannose-6-phosphate receptor, sortilin-1, and cathepsins to enter cells. Virus Research.

[26] Liu Y (2015). The Roles of Direct Recognition by Animal Lectins in Antiviral Immunity and Viral Pathogenesis. Molecules.

[27] Zhang K-Y, Yuan W-J, Xu J-D, Wang J-X (2018). Cation-dependent mannose-6-phosphate receptor functions as a pattern recognition receptor in anti-bacterial immunity of *Marsupenaeus japonicus*. Developmental & Comparative Immunology.

[28] Ferreira MJ, Lima C, Lopes-Ferreira M (2014). Anti-inflammatory effect of Natterins, the major toxins from the *Thalassophryne nattereri* fish venom is dependent on TLR4/MyD88/PI3K signaling pathway. Toxicon.

[29] Komegae EN (2011). Insights into the local pathogenesis induced by fish toxins: role of natterins and nattectin in the disruption of cell-cell and cell-extracellular matrix interactions and modulation of cell migration. Toxicon.

[30] Magalhaes G (2005). Natterins, a new class of proteins with kininogenase activity characterized from fish venom. Biochimie.

[31] Liu Y (2018). A DM9-containing protein from oyster *Crassostrea gigas* (CgDM9CP-2) serves as a multipotent pattern recognition receptor. Developmental & Comparative Immunology.

[32] Vasta, G. R. *et al*. Galectins as self/non-self recognition receptors in innate and adaptive immunity: an unresolved paradox. *Front Immunol* **3** (2012). 10.3389/fimmu.2012.00199 PMC3396283 22811679

[33] Vasta GR, Feng C, Bianchet MA, Bachvaroff TR, Tasumi S (2015). Structural, functional, and evolutionary aspects of galectins in aquatic mollusks: From a sweet tooth to the Trojan horse. Fish & Shellfish Immunology.

[34] Roux MM, Pain A, Klimpel KR, Dhar AK (2002). The Lipopolysaccharide and β-1,3-Glucan Binding Protein Gene Is Upregulated in White Spot Virus-Infected Shrimp (*Penaeus stylirostris*). J Virol.

[35] Bushkin, G. G. *et al*. Evidence for a Structural Role for Acid-Fast Lipids in Oocyst Walls of *Cryptosporidium*, *Toxoplasma*, and *Eimeria*. *mBio* **4** (2013). 10.1128/mBio.00387-13 PMC3760245 24003177

[36] Armon, R., Gold, D. & Zuckerman, U. Environmental Aspects of *Cryptosporidium*. *Journal of Veterinary Medicine and Research* **8** (2016).

[37] Samuelson J, Bushkin GG, Chatterjee A, Robbins PW (2013). Strategies To Discover the Structural Components of Cyst and Oocyst Walls. Eukaryot Cell.

[38] Kishore U (2004). Structural and functional anatomy of the globular domain of complement protein C1q. Immunology Letters.

[39] Zong Y (2019). A novel globular C1q domain containing protein (C1qDC-7) from *Crassostrea gigas* acts as pattern recognition receptor with broad recognition spectrum. Fish & Shellfish Immunology.

[40] Calandra T, Roger T (2003). Macrophage migration inhibitory factor: a regulator of innate immunity. Nature Reviews Immunology.

[41] Cui S (2011). A macrophage migration inhibitory factor like oxidoreductase from pearl oyster *Pinctada fucata* involved in innate immune responses. Fish & Shellfish Immunology.

[42] Li F (2011). A macrophage migration inhibitory factor like gene from scallop Chlamys farreri: Involvement in immune response and wound healing. Developmental & Comparative Immunology.

[43] Wang D (2018). Two macrophage migration inhibitory factors (MIFs) from the clam Ruditapes philippinarum: Molecular characterization, localization and enzymatic activities. Fish & Shellfish Immunology.

[44] Isberg RR, Tran Van Nhieu G (1994). Binding and internalization of microorganisms by integrin receptors. Trends Microbiol..

[45] Ballarin L, Scanferla M, Cima F, Sabbadin A (2002). Phagocyte spreading and phagocytosis in the compound ascidian *Botryllus schlosseri*: evidence for an integrin-like, RGD-dependent recognition mechanism. Developmental & Comparative Immunology.

[46] Terahara K (2006). Differences in integrin-dependent phagocytosis among three hemocyte subpopulations of the Pacific oyster “*Crassostrea gigas*”. Developmental & Comparative Immunology.

[47] Moreira CGA, Jacinto A, Prag S (2013). Drosophila integrin adhesion complexes are essential for hemocyte migration *in vivo*. Biol Open.

[48] Cooper, G. M. Actin, Myosin, and Cell Movement. The Cell: A Molecular Approach. 2nd edition (2000).

[49] Davis JR (2015). Inter-Cellular Forces Orchestrate Contact Inhibition of Locomotion. Cell.

[50] Olazabal IM (2002). Rho-kinase and myosin-II control phagocytic cup formation during CR, but not FcgammaR, phagocytosis. Curr. Biol..

[51] Le Foll F (2010). Characterisation of *Mytilus edulis* hemocyte subpopulations by single cell time-lapse motility imaging. Fish & Shellfish Immunology.

[52] Nikapitiya C, Dorrington T, Gomez-Chiarri M (2013). The role of histones in the immune responses of aquatic invertebrates. ISJ.

[53] Kawasaki H, Iwamuro S (2008). Potential roles of histones in host defense as antimicrobial agents. Infect Disord Drug Targets.

[54] Poirier AC (2014). Antimicrobial Histones and DNA Traps in Invertebrate Immunity: EVIDENCES IN *CRASSOSTREA GIGAS*. Journal of Biological Chemistry.

[55] Kopitar-Jerala N (2012). The Role of Cysteine Proteinases and their Inhibitors in the Host-Pathogen Cross Talk. Curr Protein Pept Sci.

[56] Ricklin D, Hajishengallis G, Yang K, Lambris JD (2010). Complement: a key system for immune surveillance and homeostasis. Nature Immunology.

[57] Peng M (2017). Expression of a novel complement C3 gene in the razor clam *Sinonovacula constricta* and its role in innate immune response and hemolysis. Developmental & Comparative Immunology.

[58] Wang L (2017). The RNA-seq analysis suggests a potential multi-component complement system in oyster *Crassostrea gigas*. Developmental & Comparative Immunology.

[59] Chen Y (2018). Molecular characterization of complement component 3 (C3) in *Mytilus coruscus* improves our understanding of bivalve complement system. Fish & Shellfish Immunology.

[60] Prado-Alvarez M, Rotllant J, Gestal C, Novoa B, Figueras A (2009). Characterization of a C3 and a factor B-like in the carpet-shell clam, *Ruditapes decussatus*. Fish & Shellfish Immunology.

[61] Romero A (2015). An immune-enriched oligo-microarray analysis of gene expression in Manila clam (*Venerupis philippinarum*) haemocytes after a Perkinsus olseni challenge. Fish & Shellfish Immunology.

[62] Green TJ, Chataway T, Melwani AR, Raftos DA (2016). Proteomic analysis of hemolymph from poly(I:C)-stimulated *Crassostrea gigas*. Fish & Shellfish Immunology.

[63] Ricklin D, Reis ES, Mastellos DC, Gros P, Lambris JD (2016). Complement component C3 – The “Swiss Army Knife” of innate immunity and host defense. Immunol Rev.

[64] Hanington PC, Zhang S-M (2010). The Primary Role of Fibrinogen-Related Proteins in Invertebrates Is Defense, Not Coagulation. J Innate Immun.

[65] Wang W, Song X, Wang L, Song L (2018). Pathogen-Derived Carbohydrate Recognition in Molluscs Immune Defense. International Journal of Molecular Sciences.

[66] Zhang H (2009). A fibrinogen-related protein from bay scallop *Argopecten irradians* involved in innate immunity as pattern recognition receptor. Fish Shellfish Immunol.

[67] Xiang Z (2014). Characteristic and functional analysis of a ficolin-like protein from the oyster *Crassostrea hongkongensis*. Fish Shellfish Immunol.

[68] Wang K, Pales Espinosa E, Tanguy A, Allam B (2016). Alterations of the immune transcriptome in resistant and susceptible hard clams (*Mercenaria mercenaria*) in response to Quahog Parasite Unknown (QPX) and temperature. Fish Shellfish Immunol.

[69] Romero A (2011). Individual sequence variability and functional activities of fibrinogen-related proteins (FREPs) in the Mediterranean mussel (*Mytilus galloprovincialis*) suggest ancient and complex immune recognition models in invertebrates. Dev. Comp. Immunol..

[70] Lean I-S, Lacroix-Lamandé S, Laurent F, McDonald V (2006). Role of Tumor Necrosis Factor Alpha in Development of Immunity against *Cryptosporidium parvum* Infection. Infect Immun.

[71] Goetz FW, Planas JV, MacKenzie S (2004). Tumor necrosis factors. Developmental & Comparative Immunology.

[72] Sun Y (2014). The immunomodulation of a novel tumor necrosis factor (CgTNF-1) in oyster *Crassostrea gigas*. Developmental & Comparative Immunology.

[73] Saeij JPJ (2007). *Toxoplasma* co-opts host gene expression by injection of a polymorphic kinase homologue. Nature.

[74] Hu G, Zhou R, Liu J, Gong A-Y, Chen X-M (2010). MicroRNA-98 and let-7 Regulate Expression of Suppressor of Cytokine Signaling 4 in Biliary Epithelial Cells in Response to *Cryptosporidium parvum* Infection. J Infect Dis.

[75] Lee J-Y, Orlikova B, Diederich M (2015). Signal Transducers and Activators of Transcription (STAT) Regulatory Networks in Marine Organisms: From Physiological Observations towards Marine Drug Discovery. Mar Drugs.

[76] Okugawa S (2013). The SOCS and STAT from JAK/STAT signaling pathway of *kuruma shrimp* Marsupenaeus japonicus: Molecular cloning, characterization and expression analysis. Molecular and Cellular Probes.

[77] Severo MS, Levashina EA (2014). Mosquito defenses against Plasmodium parasites. Current Opinion in Insect Science.

[78] Pei G, Bronietzki M, Gutierrez MG (2012). Immune regulation of Rab proteins expression and intracellular transport. Journal of Leukocyte Biology.

[79] Braun V, Niedergang F (2006). Linking exocytosis and endocytosis during phagocytosis. Biology of the Cell.

[80] Rosales C, Uribe-Querol E (2017). Phagocytosis: A Fundamental Process in Immunity. BioMed Research International.

[81] Rougerie P, Miskolci V, Cox D (2013). Generation of membrane structures during phagocytosis and chemotaxis of macrophages: role and regulation of the actin cytoskeleton. Immunol Rev.

[82] Elliott DA (2001). *Cryptosporidium parvum* Infection Requires Host Cell Actin Polymerization. Infect Immun.

[83] Lau Y-T, Gambino L, Santos B, Pales Espinosa E, Allam B (2018). Regulation of oyster (Crassostrea virginica) hemocyte motility by the intracellular parasite *Perkinsus marinus*: A possible mechanism for host infection. Fish & Shellfish Immunology.

[84] Xu, J.-D. *et al*. A Small GTPase, RhoA, Inhibits Bacterial Infection Through Integrin Mediated Phagocytosis in Invertebrates. *Front Immunol* **9** (2018). 10.3389/fimmu.2018.01928 PMC6127615 30233567

[85] Le Guernic A, Geffard A, Le Foll F, Palos Ladeiro M (2020). Comparison of viability and phagocytic responses of hemocytes withdrawn from the bivalves *Mytilus edulis* and *Dreissena polymorpha*, and exposed to human parasitic protozoa. International Journal for Parasitology.

[86] Conus S, Simon H-U (2010). Cathepsins and their involvement in immune responses. Swiss Med Wkly.

[87] Weinberg ED (1999). The Role of Iron In Protozoan and Fungal Infectious Diseases. Journal of Eukaryotic Microbiology.

[88] Sheng, J. Q. *et al*. Immunological function and antibacterial activity of two ferritin proteins from the freshwater pearl mussel *Hyriopsis schlegelii*. *Genet. Mol. Res*. **15** (2016). 10.4238/gmr.15038533 27706646

[89] Zhang Y (2013). Identification and characterization of four ferritin subunits involved in immune defense of the Yesso scallop (*Patinopecten yessoensis*). Fish & Shellfish Immunology.

[90] Zhang H (2019). Identification of two ferritin genes and their expression profiles in response to bacterial challenge in noble scallop *Chlamys nobilis* with different carotenoids content. Fish & Shellfish Immunology.

[91] Perrigault M, Tanguy A, Allam B (2009). Identification and expression of differentially expressed genes in the hard clam, *Mercenaria mercenaria*, in response to quahog parasite unknown (QPX). BMC Genomics.

[92] Sokolova IM (2009). Apoptosis in molluscan immune defense. Invertebrate Survival Journal.

[93] Bruchhaus I, Roeder T, Rennenberg A, Heussler VT (2007). Protozoan parasites: programmed cell death as a mechanism of parasitism. Trends in Parasitology.

[94] Hughes FM, Foster B, Grewal S, Sokolova IM (2010). Apoptosis as a host defense mechanism in *Crassostrea virginica* and its modulation by *Perkinsus marinus*. Fish & Shellfish Immunology.

[95] Gervais O, Renault T, Arzul I (2018). Molecular and cellular characterization of apoptosis in flat oyster a key mechanisms at the heart of host-parasite interactions. Scientific Reports.

[96] Rada B, Leto TL (2008). Oxidative Innate Immune Defenses by Nox/Duox Family NADPH Oxidases. In Contributions to Microbiology.

[97] Anderson RS (2001). Reactive Oxygen Species and Antimicrobial Defenses of Invertebrates: A Bivalve Model. In Phylogenetic Perspectives on the Vertebrate Immune System.

[98] Yang Y, Bazhin AV, Werner J, Karakhanova S (2013). Reactive Oxygen Species in the Immune System. International Reviews of Immunology.

[99] de Lorgeril J, Zenagui R, Rosa RD, Piquemal D, Bachère E (2011). Whole Transcriptome Profiling of Successful Immune Response to Vibrio Infections in the Oyster *Crassostrea gigas* by Digital Gene Expression Analysis. Plos One.

[100] Huang, J. *et al*. Hemocytes in the extrapallial space of *Pinctada fucata* are involved in immunity and biomineralization. *Scientific Reports* **8** (2018). 10.1038/s41598-018-22961-y PMC5854705 29545643

[101] Wu J (2017). The expression of superoxide dismutase in *Mytilus coruscus* under various stressors. Fish & Shellfish Immunology.

[102] Gonzalez M (2005). Evidence in oyster of a plasma extracellular superoxide dismutase which binds LPS. Biochemical and Biophysical Research Communications.

[103] Liu C (2016). The modulation of extracellular superoxide dismutase in the specifically enhanced cellular immune response against secondary challenge of *Vibrio splendidus* in Pacific oyster (*Crassostrea gigas*). Developmental & Comparative Immunology.

[104] Robalino J (2004). Induction of antiviral immunity by double-stranded RNA in a marine invertebrate. J. Virol..

[105] Jiang L (2019). Distinct Functions of Bombyx mori Peptidoglycan Recognition Protein 2 in Immune Responses to Bacteria and Viruses. Front. Immunol..

[106] Dziarski R (2004). Peptidoglycan recognition proteins (PGRPs). Molecular Immunology.

[107] Itoh N, Takahashi KG (2009). A novel peptidoglycan recognition protein containing a goose-type lysozyme domain from the Pacific oyster, Crassostrea gigas. Molecular Immunology.

[108] Wang Q, Ren M, Liu X, Xia H, Chen K (2019). Peptidoglycan recognition proteins in insect immunity. Molecular Immunology.

[109] Ni D (2007). Molecular cloning and mRNA expression of peptidoglycan recognition protein (PGRP) gene in bay scallop (*Argopecten irradians*, Lamarck 1819). Developmental & Comparative Immunology.

[110] Yang D (2019). A peptidoglycan recognition protein involved in immune recognition and immune defenses in *Ruditapes philippinarum*. Fish & Shellfish Immunology.

[111] Li JH, Chang MX, Xue NN, Nie P (2013). Functional characterization of a short peptidoglycan recognition protein, PGRP5 in grass carp *Ctenopharyngodon idella*. Fish & Shellfish Immunology.

[112] Ma P, Wang Z, Pflugfelder SC, Li D-Q (2010). Toll-like Receptors Mediate Induction of Peptidoglycan Recognition Proteins in Human Corneal Epithelial Cells. Exp Eye Res.

[113] Wang L (2019). The transcriptomic expression of pattern recognition receptors: Insight into molecular recognition of various invading pathogens in Oyster *Crassostrea gigas*. Developmental & Comparative Immunology.

[114] Jian J, Konopka J, Liu C (2013). Insights into the role of progranulin in immunity, infection, and inflammation. J Leukoc Biol.

[115] Moresco EMY, Beutler B (2011). Special Delivery: Granulin Brings CpG DNA to Toll-like Receptor 9. Immunity.

[116] Park B (2011). Granulin Is a Soluble Cofactor for Toll-like Receptor 9 Signaling. Immunity.

[117] Pila EA (2016). Endogenous growth factor stimulation of hemocyte proliferation induces resistance to *Schistosoma mansoni* challenge in the snail host. Proc. Natl. Acad. Sci. U.S.A.

[118] Hambrook JR, Gharamah AA, Pila EA, Hussein S, Hanington PC (2020). Biomphalaria glabrata Granulin Increases Resistance to Schistosoma mansoni Infection in Several Biomphalaria Species and Induces the Production of Reactive Oxygen Species by Haemocytes. Genes.

[119] Ahmad S, Hur S (2015). Helicases in Antiviral Immunity: Dual Properties as Sensors and Effectors. Trends in Biochemical Sciences.

[120] Li W, Tang X, Xing J, Sheng X, Zhan W (2014). Proteomic Analysis of Differentially Expressed Proteins in *Fenneropenaeus chinensis* Hemocytes upon White Spot Syndrome Virus Infection. PLoS One.

[121] She S (2018). Proteomics Based Identification of Autotaxin As An Anti-Hepatitis B Virus Factor and a Promoter of Hepatoma Cell Invasion and Migration. Cellular Physiology and Biochemistry.

[122] Hsia K-C, Li C-L, Yuan HS (2005). Structural and functional insight into sugar-nonspecific nucleases in host defense. Current Opinion in Structural Biology.

[123] Liu J-J, Sturrock R, Ekramoddoullah AKM (2010). The superfamily of thaumatin-like proteins: its origin, evolution, and expression towards biological function. Plant Cell Rep.

[124] Niu X (2018). The antifungal activity of a thaumatin-like protein from oyster *Crassostrea gigas*. *Invertebrate Survival*. Journal.

[125] Lieleg O, Lieleg C, Bloom J, Buck CB, Ribbeck K (2012). Mucin biopolymers as broad-spectrum antiviral agents. Biomacromolecules.

[126] Renault T (2015). Immunotoxicological effects of environmental contaminants on marine bivalves. Fish & Shellfish Immunology.

[127] Delaporte M (2003). Effect of a mono-specific algal diet on immune functions in two bivalve species - *Crassostrea gigas* and *Ruditapes philippinarum*. Journal of Experimental Biology.

[128] Belkaid Y, Hand TW (2014). Role of the Microbiota in Immunity and Inflammation. Cell.

[129] Liu, Z., Li, M., Yi, Q., Wang, L. & Song, L. The Neuroendocrine-Immune Regulation in Response to Environmental Stress in Marine Bivalves. *Front Physiol* **9** (2018). 10.3389/fphys.2018.01456 PMC6282093 30555334

[130] Nguyen TV, Alfaro AC (2020). Applications of omics to investigate responses of bivalve haemocytes to pathogen infections and environmental stress. Aquaculture.

[131] Tsakas S, Marmaras VJ (2010). Insect immunity and its signalling: an overview. Invertebrate Survival Journal.

[132] Venier, P., Domeneghetti, S., Sharma, N., Pallavicini, A. & Gerdol, M. Chapter 7 - Immune-Related Signaling in Mussel and Bivalves. In Lessons in Immunity (eds. Ballarin, L. & Cammarata, M.) 93–105 (2016).

[133] Hartmann EM, Allain F, Gaillard J-C, Pible O, Armengaud J (2014). Taking the Shortcut for High-Throughput Shotgun Proteomic Analysis of Bacteria. In Host-Bacteria Interactions: Methods and Protocols.

[134] Klein G (2016). RNA-binding proteins are a major target of silica nanoparticles in cell extracts. Nanotoxicology.

[135] Carvalho PC (2016). Integrated analysis of shotgun proteomic data with PatternLab for proteomics 4.0. Nature Protocols.

[136] Marchler-Bauer A (2011). CDD: a Conserved Domain Database for the functional annotation of proteins. Nucleic Acids Research.

[137] Perez-Riverol Y (2019). The PRIDE database and related tools and resources in 2019: improving support for quantification data. Nucleic Acids Res.

